# From Joint Clinical Assessment to National Decisions: EU HTA Implementation and Implications for Patient Access

**DOI:** 10.3390/jmahp14030050

**Published:** 2026-08-19

**Authors:** Kalpana D’Oca, Ada Adriano, Eline Darquennes, Natalie Steck

**Affiliations:** 1 MSD (UK) Limited, London EC2M 6UR, UK; ada.adriano@msd.com; 2 MSD Belgium, 1170 Brussels, Belgium; eline.darquennes@msd.com; 3 MSD Innovation & Development GmbH, CH-8058 Zurich, Switzerland; natalie.steck@msd.com

**Keywords:** EU HTA, JCA, PICO scoping, national reimbursement timelines

## Abstract

The introduction of Joint Clinical Assessments (JCAs) under the European Union Health Technology Assessment Regulation (EU HTAR) represents a major structural reform aimed at reducing fragmentation and duplication in clinical evidence assessment across Member States. While JCAs are intended to support national health technology assessment (HTA) processes through a common EU-level clinical evaluation, their integration into established national HTA and reimbursement systems remains untested at this early stage of implementation. This paper reports findings from two surveys conducted in 2025 among local affiliate representatives with expertise in relevant HTA and market access activities across 25 European countries (comprising 24 EU Member States and Norway), capturing early national perspectives on key aspects of JCA implementation. The surveys explored anticipated impact on national reimbursement timelines, current opportunities for early HTA advice and PICO input, approaches to handling post-JCA data availability, and nationally prioritised policy and implementation issues. Results indicate mixed expectations regarding the potential impact of JCA on reimbursement timelines, with perceived risks of delay largely viewed as possibly transitional and mitigable through national process adaptation. Respondents emphasised the importance of transparency in PICO consolidation and clarity on the use of JCA reports in national appraisals. Overall, the findings suggest that JCA may represent a reconfiguration rather than a centralisation of evidence assessment, with national influence exercised earlier in the assessment lifecycle. These insights provide policy-relevant input to inform ongoing EU HTA implementation and future refinement of the JCA framework.

## 1. Introduction

Fragmentation across EU Member States has previously been identified as a key structural weakness affecting pharmaceutical competitiveness, arising from heterogeneous national health technology assessment (HTA), pricing and reimbursement processes [1,2]. In innovation-intensive sectors such as the pharmaceutical industry, this can hinder the rapid market deployment of new innovations by incurring both increased costs and delays. In practice, this may translate into duplicated assessments, requests to address divergent evidence requirements, and misaligned timelines across HTA and reimbursement processes, thereby undermining Europe’s ability to translate pharmaceutical innovation into timely patient access [2].

The introduction of Joint Clinical Assessments (JCAs) under the EU Health Technology Assessment Regulation (EU HTAR) aims to address such inefficiencies, representing a significant structural shift toward harmonised evaluation of clinical effectiveness and safety evidence across Member States [3,4]. By providing a single, harmonised EU-level clinical assessment that aims to reduce duplication and improve methodological consistency and coordination across Member States, as well as strengthen alignment between regulatory evaluation and national HTA processes, JCA has the potential to enhance predictability and efficiency in access pathways. This supports the ambition of the EU HTAR to facilitate “*timely and informed decisions on the pricing and reimbursement of health technologies*” [3] at a national level, which will contribute to “*faster and wider access to new and more effective innovative products for patients*” [3].

The JCA report, produced at the conclusion of the assessment process, provides a scientific analysis of the clinical evidence on the relative effects of a health technology on health outcomes [5]. It is intended to serve as an input to national HTA processes, providing a common clinical assessment across Member States. However, JCAs do not restrict Member States’ ability to make context-specific decisions, thereby preserving national competence over health policy, pricing, reimbursement, and value determination. National HTA therefore remains the contextualisation and decision-making layer in which evidence is interpreted in light of country-specific socio-economic factors.

Under the EU HTAR, Member States are required to give “*due consideration*” [6,7] to JCA reports. Nevertheless, considerable uncertainty remains regarding how this requirement will be operationalised within national HTA decision-making [8]. Anticipated approaches range from limited reference to, or annexation of, the JCA report to its full incorporation as the clinical component of national submissions [8]. These differing approaches raise questions about how national contextual evidence requirements will be addressed in practice. In addition, jurisdictions using cost-effectiveness frameworks will continue to conduct their local economic evaluations based on country-specific inputs [9].

Although JCAs are designed to standardise clinical evaluation across the EU, tensions may persist with national HTA systems that focus on locally defined PICOs and reimbursement criteria, unless efforts are made to ensure that JCA outputs remain relevant at a national level and can meaningfully inform national assessments [4,10]. It is critical to consider how JCA will be integrated into national assessments, as insufficient alignment risks increased complexity and delays rather than efficiency gains. At this early stage of implementation, the real-world impact of JCA on national reimbursement timelines cannot yet be reliably assessed. Previous research [11] has considered the potential impact of JCA being a new requirement to inform local HTAs. Understandably, assessments concerning the impact of JCA are anticipatory in nature and forward-looking, given the regulation is in its early implementation phase and empirical evidence on national uptake and decision-making remains limited [12]. Meaningful evaluation will require completion of multiple JCA procedures followed by downstream national HTA and reimbursement decisions, after which systematic trends may begin to emerge. It has been acknowledged that further observation is necessary to remain up to date with evolving perspectives from all relevant stakeholders, as we move forward through the initial implementation phase [11].

The objective of this manuscript is to contribute to the existing body of literature by highlighting local perspectives and expectations on the early implementation of JCA under the EU HTAR, with particular focus on the anticipated interaction between EU-level assessment processes and established national HTA frameworks. To achieve this, we sought feedback from local company affiliates on anticipated implications for reimbursement timelines and evidence requirements, current national approaches to PICO-related activities and scientific advice, and policy and implementation topics perceived as priorities by local affiliate stakeholders, which may warrant further focus to inform future refinement and evaluation of the JCA framework.

## 2. Methods

Two independent surveys were conducted, as described below. Respondents were country-level local affiliate company representatives with responsibilities and/or subject-matter expertise in national HTA, pricing and reimbursement, market access, or related policy functions, covering perspectives from 25 European countries (comprising 24 EU member states, plus Norway). These countries were selected to reflect European markets with established HTA frameworks expected to be relevant to JCA implementation. Respondents were identified through the company’s internal regional and country affiliate networks based on their knowledge of national HTA procedures and anticipated EU HTA implementation. Responses therefore reflect informed company-affiliate perspectives, rather than formal positions of national HTA agencies or payers. Given the early stage of EU HTA implementation, several survey questions required respondents to provide informed expectations and best-estimate assessments in the absence of observed implementation outcomes.

### 2.1. Survey 1

A structured cross-sectional survey was developed to collect early national perspectives from local affiliates as described above, during the second half of 2025. The survey questionnaire was first distributed by email, allowing local affiliates time to review the items and consider their responses. Within the subsequent one- to two-month period, an individual virtual meeting was held with the affiliates. During these sessions, survey questions were discussed sequentially, and responses, including additional context, clarifications, and qualitative insights, were documented directly in an online version of the survey. Given the early stage of EU HTA implementation and the lack of availability of precedents for JCAs at the time of data collection, several questions required affiliates to provide informed assumptions or best-estimate judgments.

The survey questions focused on touchpoints of national influence across the JCA lifecycle and included items covering the following domains: (1) anticipated effects of the JCA on national HTA submission and reimbursement timelines and (2) procedural interdependencies between the JCA process and national HTA requirements. Details of the survey questions and response options are provided in Supplementary Material. Closed-ended responses were summarised descriptively, with findings reported as frequencies and percentages (*n*, %), where appropriate. Free-text responses and contextual information collected during affiliate discussions were reviewed and grouped into recurring themes using a descriptive thematic summarisation approach. Themes were retained where they recurred across multiple country-affiliate discussions or provided relevant explanatory context for quantitative findings. No formal qualitative coding framework or inter-coder reliability assessment was applied; therefore, qualitative findings should be interpreted as descriptive contextual summaries to support interpretation of the quantitative results.

### 2.2. Survey 2

A separate survey was sent to the local affiliates during the second half of 2025, asking them to prioritise, from a pre-defined list, the policy-related issues they considered most relevant from their national perspectives. The list of issues was developed by the European Regional team dedicated to EU HTA preparation based on topics identified as key inputs for a planned update of the company’s EU HTA position paper. The issues presented for ranking, listed alphabetically, were:

- Consideration of the JCA report in national appraisals and the impact of delays in the JCA process;
- Development of a dedicated JCA process for vaccines;
- European Commission support for patient organisations;
- Submission of additional data packages to the EMA and associated notification obligations;
- Systematic stakeholder feedback, including industry input, on the JCA process to inform its evaluation by 2028;
- Transparency criteria for PICO consolidation.

Local affiliates were invited to respond to the survey within a 2-week window. Responses were subsequently consolidated and ranked in order of importance.

During the preparation of this manuscript, the authors used Microsoft 365 Copilot (Microsoft 365 2607) for the purpose of editing certain text and to support the generation of figure.

## 3. Results

Percentages are based on the number of country perspectives available for the relevant survey item, based on responses received from local affiliates. For Survey 1, responses representing 25 of 25 country perspectives were available. For Survey 2, responses from local representatives representing 19/25 countries surveyed were received with their ranked prioritisation of policy issues, representing a 76% response rate; therefore, percentages presented for this analysis use 19 as the denominator.

Aggregate findings from the surveys are presented below. A selective anonymisation approach was applied. Country-level findings were reported where these related to identifiable characteristics of national HTA processes (e.g., availability of scientific advice or opportunities for PICO-related engagement). In contrast, findings reflecting respondent expectations, anticipated implementation approaches, or policy-related perspectives were reported in aggregated or anonymised form to avoid attributing forward-looking positions to specific countries, represented by local affiliate perspectives, during the early stages of JCA implementation.

### 3.1. Findings from Survey 1

#### 3.1.1. Anticipated Impact of JCA on Timeline to National Reimbursement Decision

**Figure 1:**
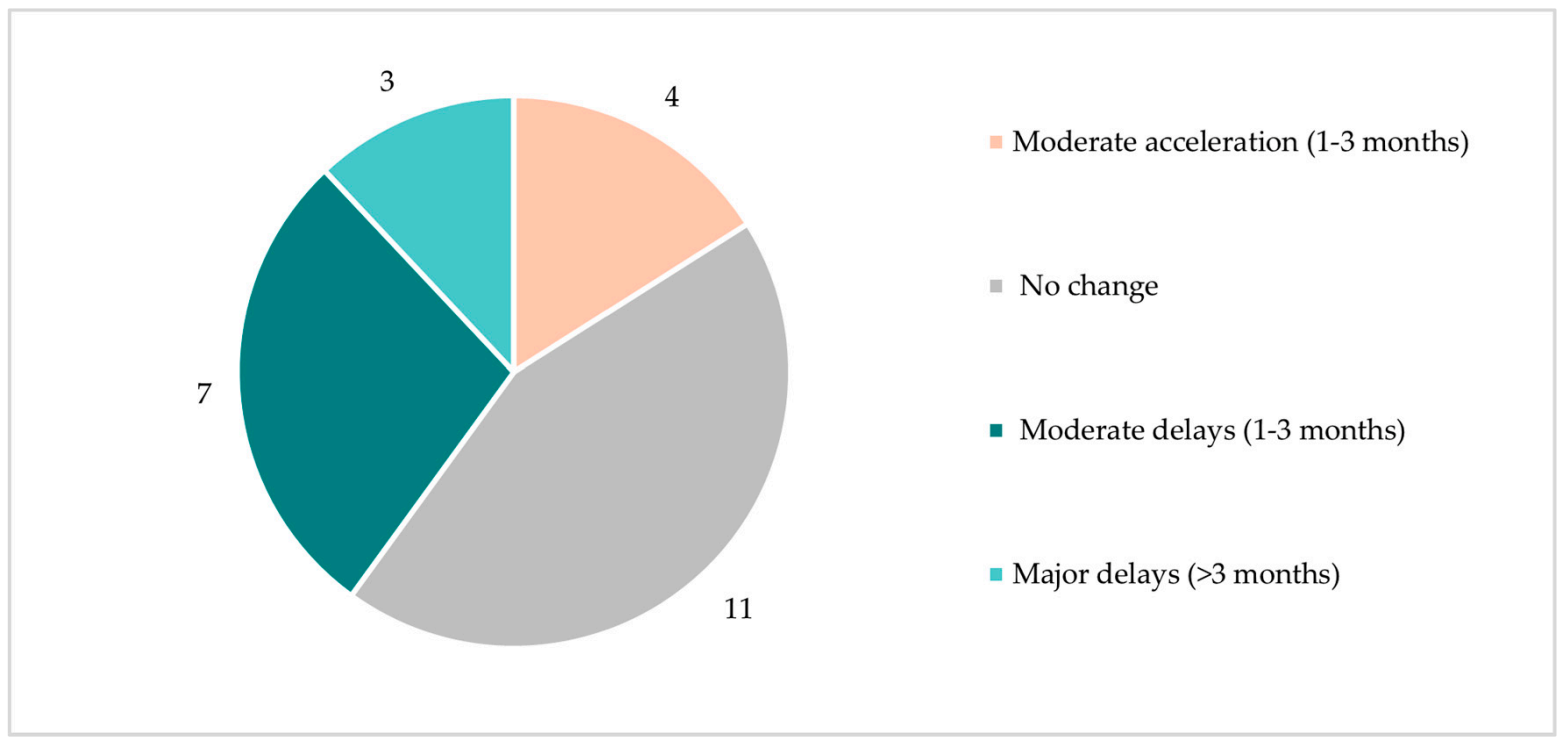
Anticipated impact of JCA on national reimbursement timeline.

Of the local affiliate perspectives collected, 56% (14/25) reported an expectation that JCA may influence the timeline to national reimbursement decision, either accelerating the time to reimbursement decision or causing a delay, while 44% (11/25) anticipated that the introduction of JCA would be unlikely to have a net impact on the timeline to national reimbursement decision (Figure 1). Responses from the 40% (10/25) of local affiliate respondents who reported an expectation or concern that JCA implementation could contribute to delays in national reimbursement decision timelines identified several interrelated factors that were perceived as potential drivers of such delays. The expectation that national HTA submissions, or assessment initiation, would occur only after publication of the JCA report rather than at earlier regulatory milestones was cited as a factor potentially introducing delays of approximately one to three months to national submissions, with downstream implications for the timeline to reimbursement decision. However, it was also highlighted that this impact may be partially mitigated in practice, as national HTA dossiers are already typically submitted within a few months of EMA approval rather than earlier at CHMP opinion, thereby limiting incremental delays in some settings.

Overall, respondents emphasised that the magnitude of impact on timelines to national reimbursement decision remains unclear, with responses reflecting anticipated risks rather than observed delays, given that no products had yet completed the full JCA-to-national reimbursement pathway at the time of data collection. Uncertainty regarding procedural requirements, such as whether national dossier completeness checks would depend on availability of the JCA report, was also perceived as a source of potential delay, particularly during early implementation. Capacity and resourcing constraints within HTA agencies were cited as an important compounding factor, with some respondents anticipating delays of up to four months due to increased workload, learning effects, and broader system pressures affecting both JCAs and non-JCA submissions. These capacity-related delays were further expected to have knock-on effects for pricing, budget allocation processes and ultimately patient access, particularly where delayed assessment timelines could preclude inclusion in annual budget cycles.

Of the 40% (10/25) of local affiliates reporting potential concerns about reimbursement delays associated with JCA, there was universal belief that opportunities exist to adapt national systems to mitigate such delays, mainly through continuous engagement between trade associations and HTA agencies.

#### 3.1.2. Current Opportunities for National Early HTA Advice to Inform Phase III (Pivotal) Study Design

Among the local affiliate perspectives collected, 20% (5/25: France, Germany, Italy, The Netherlands and Sweden) indicated that prior to the implementation of JCA, opportunities already existed to seek advice from their national HTA body to inform Phase III (pivotal) clinical trial design. Feedback representing one additional country (Spain) suggested that a similar mechanism was expected to be introduced in the near future following the then anticipated publication of new Royal Decree legislation. Joint Scientific Consultations (JSC), introduced under the EU HTAR, offer a valuable new opportunity to obtain coordinated, pan-European input on key trial design considerations, including PICO elements relevant to technologies that may be subject to a future JCA. However, feedback indicated that requesting a JSC may limit the ability to obtain parallel national-level HTA advice in some settings. As such, it was highlighted that strategic planning of JSCs should take into account the needs of countries where national advice mechanisms are currently available, but may be displaced if JSC is leveraged.

#### 3.1.3. Acquiring Feedback on PICOs of Relevance to Inform National HTA Submission Preparation

**Figure 2:**
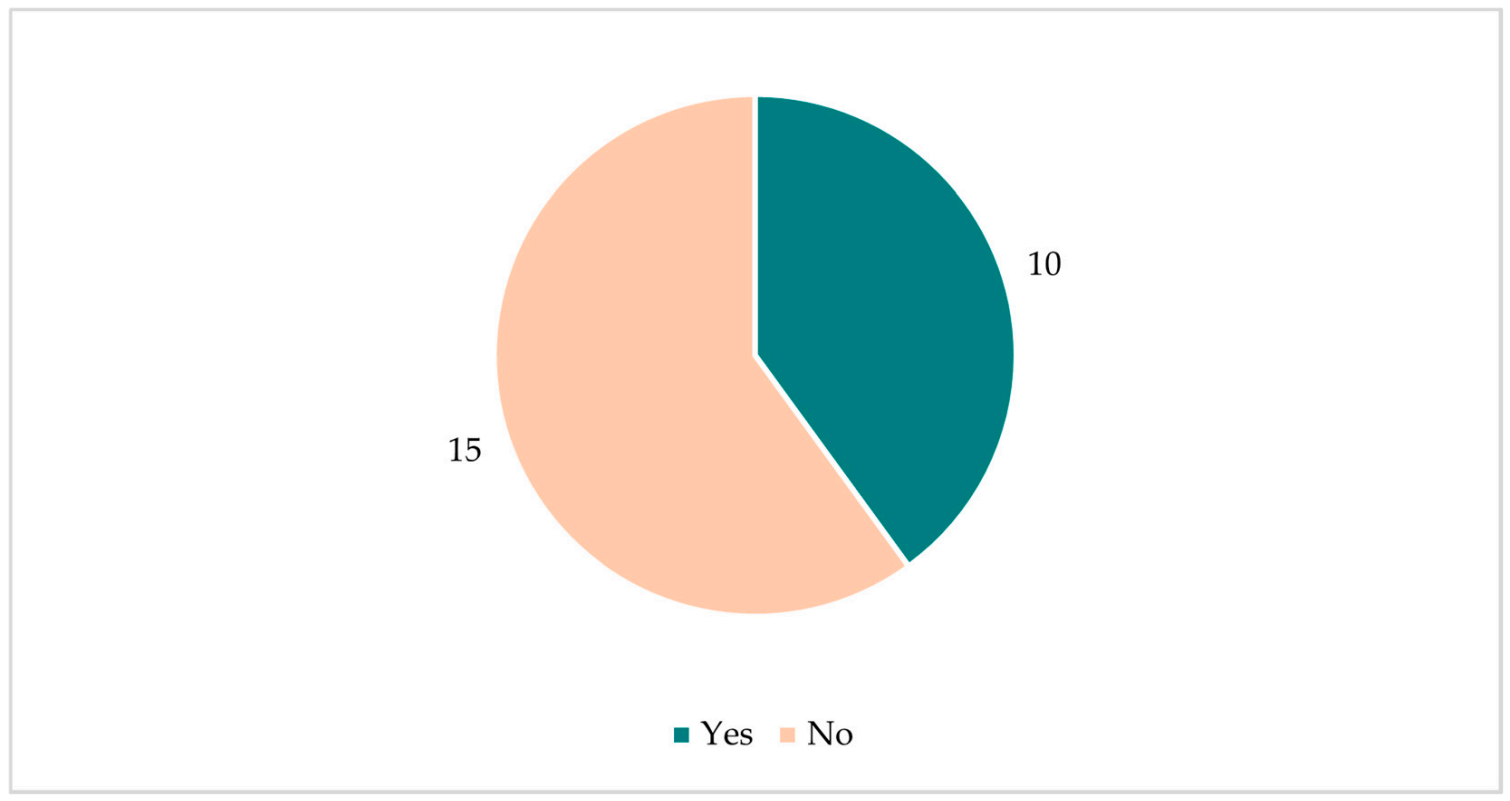
Opportunity to seek advice from HTA body about the PICOs of relevance in advance of submission.

Perspectives were requested to understand the number of countries that currently have an opportunity to seek advice or acquire feedback, either via a formal process step or through ad hoc engagement, on PICOs of relevance to inform national HTA submission preparation. Based on feedback provided, local affiliates representing 36% of included countries (9/25: Bulgaria, Czechia, Denmark, Ireland, Lithuania, Germany, The Netherlands, Norway and Poland) reported an opportunity to seek feedback from their national HTA agency about the PICOs of relevance from a national perspective. Again, feedback from one additional country (Spain) suggested that a similar mechanism was expected to be introduced following the then anticipated publication of new Royal Decree legislation (Figure 2).

#### 3.1.4. Data Availability

**Figure 3:**
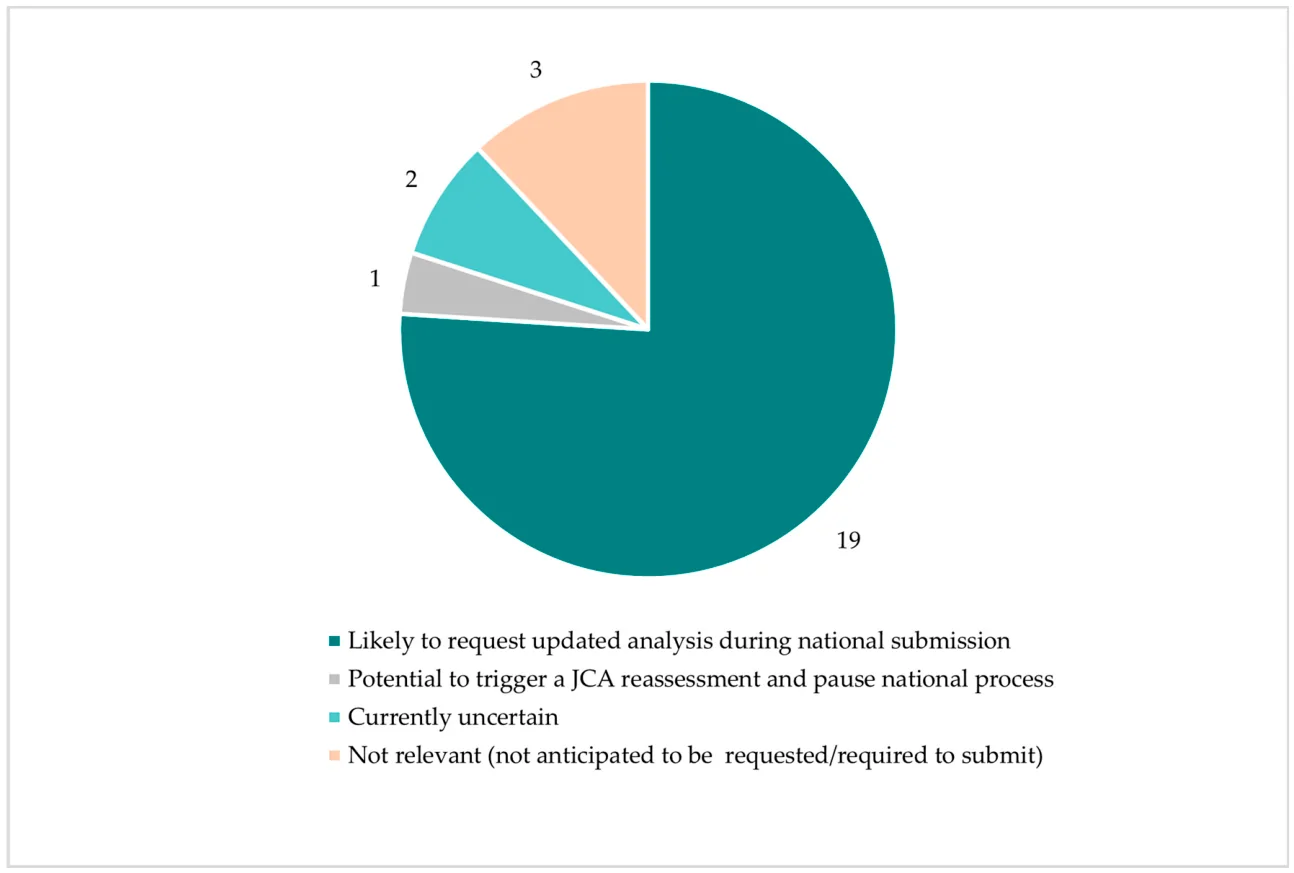
Anticipated view on how national HTA body will deal with new data cuts becoming available after JCA report publication.

To explore key areas of residual uncertainty, the survey included items assessing the anticipated implications of clinical evidence becoming available after completion of the JCA. Perspectives on the anticipated view of how national HTA bodies will deal with new data availability post publication of the JCA report are depicted below (Figure 3). While there is no certainty, most countries predicted it will be unlikely that a new data cut would trigger a JCA reassessment. The expectation is that national HTA bodies will be pragmatic and incorporate newly available data into their national assessment process, although one respondent flagged that a JCA reassessment may be a possibility, which would inevitably lead to delays in national access.

### 3.2. Findings from Survey 2

#### Policy Issues Ranked in Order of Importance from a National Perspective Across European Markets

**Figure 4:**
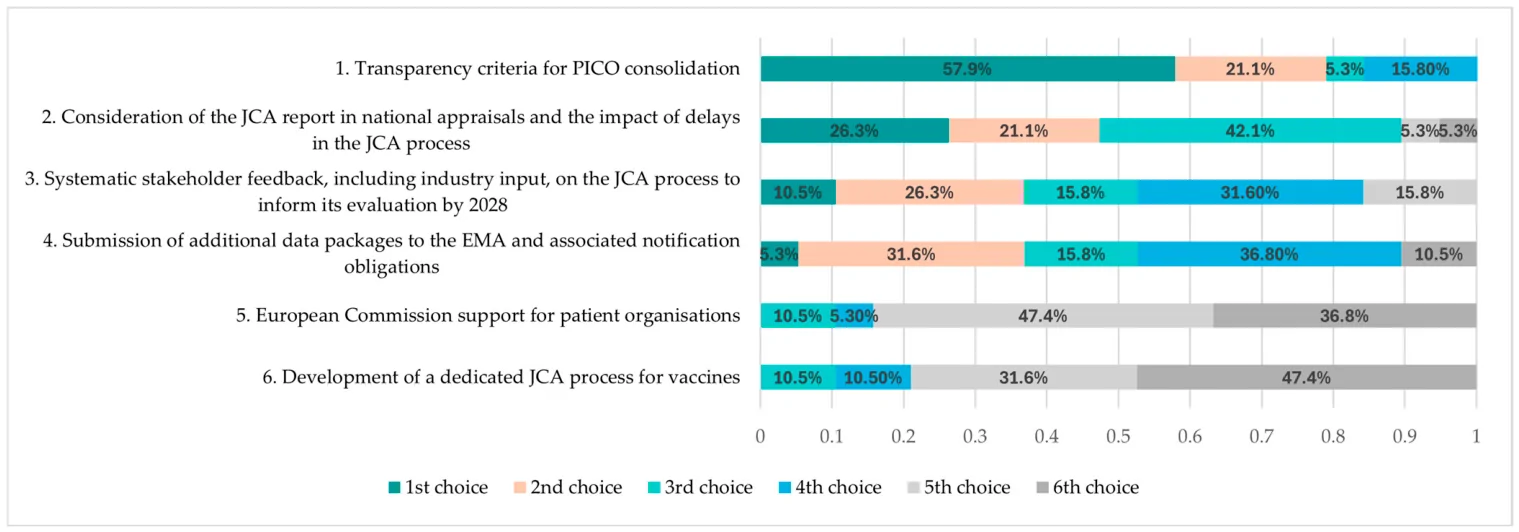
EU HTA-related policy topics in ranked order of importance from a national perspective.

Local affiliate perspectives representing 76% (19/25) of included countries were provided in response to the survey in the second half of 2025. The responses (Figure 4) provided a clear ranking of the issues considered most important from a national perspective, leading with the issue of transparency criteria for PICO consolidation, followed by the consideration of the JCA report in national appraisals and the impact of delays in the JCA process.

## 4. Discussion

The findings presented provide an interpretation of national perspectives on JCA within the context of early implementation, where expectations necessarily reflect anticipatory views (rather than observed outcomes) due to limited real-world experience.

### 4.1. Anticipated Impact of JCA on National Reimbursement Timelines and System Adaptation

The survey findings suggest that JCA is expected to have the potential to influence national reimbursement timelines in a number of Member States. Key drivers for anticipated delays included a perceived dependence on availability of the JCA report before commencement of national submission and assessment processes, procedural uncertainty during the current early implementation phase as national systems adapt to new procedural requirements, and capacity/resourcing constraints within national HTA agencies. Previous research [13] shows that as of late 2025, all 10 EU countries with the highest nominal Gross Domestic Product (GDP) had either already put in place formal guidance, or were actively drafting policies, to align dossier content, process, and timelines with the JCA, which is encouraging. We interpret respondents’ anticipated delay-related concerns identified in Survey 1 as potentially reflecting a period of system adaptation and familiarisation during the early implementation of JCA. However, this interpretation requires confirmation once products have progressed through the full JCA-to-national appraisal and reimbursement pathway. The EU HTAR does not preclude Member States from initiating national HTA procedures while a JCA is ongoing, should they choose to do so [14]. However, the timing and manner in which JCA outputs are incorporated into national decision-making are likely to vary across Member States, reflecting differences in when national HTA processes are initiated [14]. Clarity regarding the intended use of JCA reports in national appraisal procedures was ranked as the second-highest priority topic in our topic-ranking exercise, suggesting that respondents place considerable importance on understanding how JCA reports may be used within national appraisal processes, and that additional transparency could help support predictability during the early implementation phase. In rapidly evolving therapeutic areas, the importance of minimising the lag between JCA completion and initiation of national appraisal processes will be essential, as delays may reduce the relevance of the JCA output if the standard of care changes, thereby necessitating additional evidence generation and submission at national level.

Despite concerns about potential delays, local affiliates consistently reported confidence in their ability to meaningfully shape their national process at a local level to minimise impact (primarily via the local trade association route), signalling retained national influence and indicating that JCA reconfigures rather than diminishes or removes national agency in HTA decision-making.

### 4.2. Early Advice and National Coordination: Strategic Implications

Currently, there is limited, but nonetheless meaningful, availability of national early scientific advice processes across European countries to inform Phase III clinical trial design. This has important implications from a planning perspective and underscores the need for focused and strategic coordination between national advice mechanisms and JSCs, in order to maximise the opportunity for informed evidence planning. As JSC participation may, in some countries (e.g., France), displace the opportunity for national advice [15], there must be early consultation and alignment with local affiliates to facilitate the strategic sequencing of EU-level and national advice, when considered necessary, for products expected to be subject to JCA.

### 4.3. PICO Scoping, Transparency, and Alignment with National HTA Needs

In the topic ranking exercise, respondents clearly prioritised transparency in the PICO consolidation process as the area where greater predictability and trust are needed within the evolving JCA framework. The current ambiguity on this topic is consistent with previous work [16] demonstrating substantial variability in the number of PICOs generated through simulation exercises, with corresponding increases in analytical burden, reinforcing the need for greater clarity on how consolidation is undertaken in practice. Against this backdrop, our survey finding indicating that 36% of countries currently offer opportunities for national-level feedback on PICOs is noteworthy, as it illustrates that structured early dialogue on evidence expectations is already embedded within several national HTA systems and is valued in supporting preparedness for national submissions.

Increasing the level of engagement and transparency during the development of the assessment scope for JCA has been previously called for across industry [17]. This approach is not without precedent in other jurisdictions [18], where broad stakeholder input is routinely sought. This could facilitate earlier alignment on evidence requirements and reduce avoidable inefficiencies. Given challenges previously highlighted in PICO scoping, particularly around consolidation [19], procedural adaptation that improves transparency would be welcomed. At minimum, enabling Member States’ HTA agencies to share their national PICO requirements with HTDs once the JCA assessment scope has been finalised would support timely development of aligned evidence packages and economic models, while maintaining open communication channels to address subsequent changes to PICO elements that may affect national HTA submissions [9]. Without early PICO sharing and coordination, there is increased risk that national HTA submissions, including cost-effectiveness models where required, are built addressing a non-relevant set of PICOs for a particular market [9]. This will likely increase workload and contribute to further delays for all parties involved at a local level, hampering the integration of JCA in the national HTA process.

Together, the feedback from the topic ranking exercise and survey point towards the importance of early dialogue with HTDs as being critical to the effective integration and long-term credibility of JCAs. Yet the importance of opportunities for dialogue between the HTA CG and Assessors/Co-Assessors with the HTD extends beyond discussions focused solely on the PICO scoping; such dialogue would be essential from the pre-submission phase onwards and throughout the JCA process to facilitate timely clarification of key procedural and methodological issues across the end-to-end JCA process.

With its aim of reducing duplication and minimising redundancy while speeding up time to patient access, it is apparent that frequent JCA reassessments due to availability of new data would be highly impractical. Based on the findings earlier summarised, expectations that new data will be handled pragmatically at national level reflect a preference for continuity in assessment processes, with JCA reassessment viewed as an exception rather than the norm. If this transpires, it will signal a degree of flexibility in the national systems. Alternatively, if the minority view becomes reality with JCA reassessments being the elected route in the event of additional data availability, the inevitable consequence will be delays in patient access.

### 4.4. Ensuring Relevance of EU HTA Processes (JSC and JCA) for National HTA

**Figure 5:**
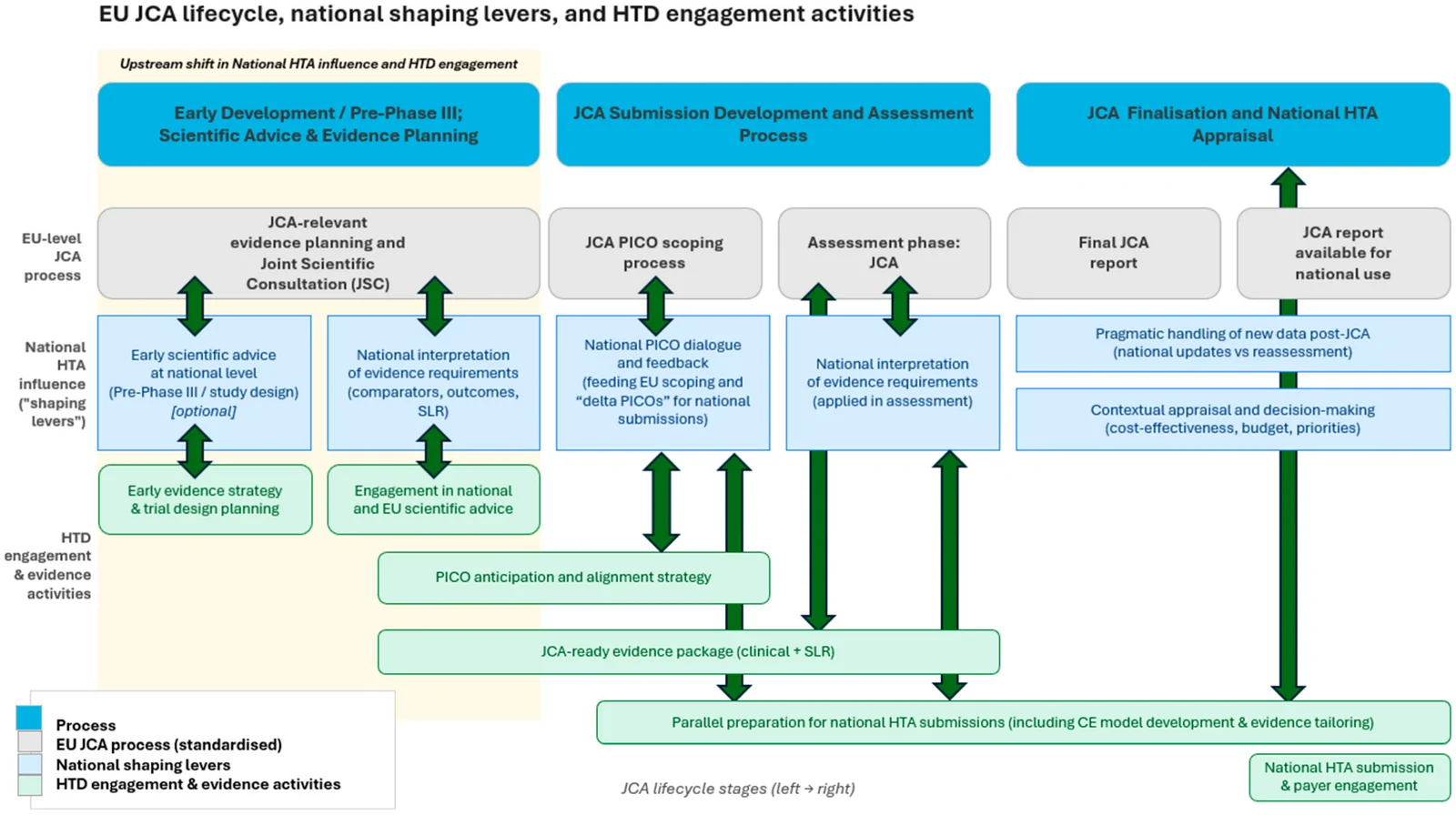
Conceptual framework of the EU JCA lifecycle, national shaping levers, and HTD engagement activities. Microsoft 365 Copilot (Microsoft 365 2607) was used to support the generation of Figure 5.

The introduction of JCAs means that national influence shifts earlier, exercised through a variety of strategic touchpoints across the JCA lifecycle. These include opportunities for early scientific advice to inform study design, national dialogue on PICO relevance with potential to feed into JCA scoping and post-JCA national appraisal processes incorporating contextual and economic considerations. Similarly, effective HTD engagement shifts upstream, requiring earlier alignment of evidence strategies with anticipated JCA and national PICO expectations, proactive engagement with scientific advice mechanisms, and preparation for parallel national processes following JCA report publication. Collectively, this conceptualisation (Figure 5) highlights that JCA brings about a structural reconfiguration of evidence preparation rather than centralising decision-making.

JSCs provide an important mechanism to inform evidence planning. Early understanding of PICO-related considerations and a consolidated view of clinical evidence needs (including their implications for cost-effectiveness analyses and modelling requirements in markets where this is a consideration) would support more coherent evidence planning and reduce misalignment between EU-level assessment and national appraisal needs. As such, JSCs have the potential to function as an upstream coordination mechanism, supporting the generation of evidence that is more readily translatable into national HTA processes. However, the current design and implementation of JSC raise important questions regarding proportionality and accessibility. Substantial cross-functional internal resourcing and co-ordination is required from the HTD to undertake JSC, in particular if parallel JSC with the EMA is sought [20]. Although JSC participation necessitates significant preparatory efforts from HTDs, limited capacity means that these efforts do not always result in access to a JSC, as opportunities are concentrated among a small subset of products [21,22]. Collectively, these considerations suggest that for JSCs to fulfil their intended role as an enabling component of the EU HTA framework, greater attention should be given to ensure proportional preparation requirements and increased capacity to improve accessibility.

## 5. Conclusions

The success of JCA should not be judged solely by procedural implementation or compliance with regulatory timelines, but by the extent to which it delivers relevant, usable, and efficiency-enhancing inputs for national HTA decision-making. The findings presented highlight that JCA represents a structural reconfiguration of clinical evidence assessment in Europe, with the potential to reduce duplication and improve coordination, while preserving national competence over appraisal, pricing, and reimbursement decisions. Realising these benefits, however, will depend on how effectively JCA outputs are integrated into national processes and translated into context-specific decision frameworks.

From an HTD perspective, the findings underscore the importance of early and sustained engagement, proactive anticipation of PICO requirements, and parallel planning across EU-level and national HTA pathways. Clear and transparent mechanisms for dialogue, particularly concerning PICO scoping and early advice, are critical to enabling the timely development of evidence packages and economic models that meet national HTA needs and minimise avoidable delays to patient access. Procedural clarity, transparency, and pragmatic implementation approaches may also support HTA bodies by improving predictability and consistency.

At this stage, the real-world impact of JCA on national access timelines cannot yet be fully assessed, as implementation remains in its early phases. It remains uncertain whether anticipated implementation challenges will be transitional or more persistent. The survey findings suggest that respondents expected some perceived risks to be mitigable through national process adaptation, but this should be interpreted as an early expectation rather than a demonstrated outcome. Proactive engagement, transparency, and timely coordination across JCA and national HTA procedural steps are likely to be important factors needed to facilitate the effective implementation of JCA and support timely patient access.

Taken together, the early empirical insights presented here contribute to the emerging evidence base on EU HTA implementation and provide policy-relevant input for ongoing discussions. As the JCA framework continues to mature, these findings may help inform the planned 2028 review of the EU HTAR [6], supporting further refinement and clarification of guidance to ensure that JCA achieves its intended objectives of efficiency, predictability, and meaningful support for national decision-making and patient access.

## 6. Strengths and Limitations

The findings provide timely insight into national expectations during the early implementation phase of JCA, offering a multi-country perspective on how JCA is anticipated to influence national processes. A key strength lies in its focus on underlying mechanisms and expectations, such as anticipated timeline impacts, opportunities for process adaptation, and areas of residual uncertainty, at a point when empirical evidence is still limited. However, the findings are inherently anticipatory in nature and reflect expected rather than observed impacts, as no products have yet to complete the full pathway from JCA through to national appraisal and reimbursement. Consequently, the decision to present some results as aggregated, anonymised perspectives is considered reasonable given the levels of uncertainty concerning the downstream national impact of JCA in the current early implementation phase. Survey findings should also be interpreted in the context of the respondent population and survey designs. Respondents were local affiliate representatives of our company and therefore provided informed industry perspectives on national EU HTA implementation rather than the views of HTA agencies, payers, clinicians or patient representatives. In addition, the list of policy-related issues included in the ranking exercise was developed by our company’s regional EU HTA team and may therefore reflect priorities currently considered relevant from an industry perspective. While these factors do not diminish the value of the finding as an early descriptive assessment of implementation expectations, they should be considered when interpreting the findings.

As the policy-priority ranking exercise achieved a response rate below 100% (76%), the possibility of non-response bias cannot be excluded, and priorities among non-responding local affiliates may have differed from those reported. The absence of real-world outcomes limits the ability to draw conclusions regarding the actual effects of JCA on access timelines or decision-making. Future observation will be needed to complement these early insights with longitudinal analyses once sufficient numbers of technologies have progressed through the full process of JCA followed by national reimbursement pathways, enabling systematic assessment of realised impacts over time.

## Data Availability

The original contributions presented in this study are included in the article. Further inquiries can be directed to the corresponding author.

## Supplementary Materials

The following supporting information can be downloaded at: https://www.mdpi.com/article/10.3390/jmahp14030050/s1, Survey S1 and S2: Issued via MS Forms in second half of 2025.

## Abbreviations

The following abbreviations are used in this manuscript:

CHMP	Committee for Medicinal Products for Human Use
EMA	European Medicines Agency
EU	European Union
EU HTA	European Health Technology Assessment
EU HTAR	European Health Technology Assessment Regulation
GDP	Gross Domestic Product
HTA	Health Technology Assessment
HTD	Health Technology Developer
JCA	Joint Clinical Assessment
JSC	Joint Scientific Consultation
PICO	Population, Intervention, Comparator, Outcomes
