# Supplementary material for: From Joint Clinical Assessment to National Decisions: EU HTA Implementation and Implications for Patient Access
Source: J Mark Access Health Policy. 2026 Aug 19;14(3):50. doi: 10.3390/jmahp14030050 (PMC13510652; doi:10.3390/jmahp14030050)
Supplement: Supplementary file 1 [file jmahp-14-00050-s001.zip › jmahp-4412313-supplementary.pdf]

Supplementary Appendix 1:

Survey 1: Issued via MS Forms in second half of 2025

Survey issued to local affiliates representing the following markets (n=25)	Responses received from local affiliates representing the following markets (n=25)	Participation rate
Austria<br>Belgium<br>Bulgaria<br>Croatia<br>Czechia<br>Denmark<br>Estonia<br>Finland<br>France<br>Germany<br>Greece<br>Hungary<br>Ireland<br>Italy<br>Latvia<br>Lithuania<br>Netherlands<br>Norway<br>Poland<br>Portugal<br>Romania<br>Slovakia<br>Slovenia<br>Spain<br>Sweden	Austria<br>Belgium<br>Bulgaria<br>Croatia<br>Czechia<br>Denmark<br>Estonia<br>Finland<br>France<br>Germany<br>Greece<br>Hungary<br>Ireland<br>Italy<br>Latvia<br>Lithuania<br>Netherlands<br>Norway<br>Poland<br>Portugal<br>Romania<br>Slovakia<br>Slovenia<br>Spain<br>Sweden	25/25 (100%)

Questions and Response Options

Impact of JCA on local HTA submission and reimbursement timelines

(1) Please select the (expected) impact of JCA on **overall reimbursement** timeline
- Major acceleration (> 3 months)
- Moderate acceleration (1-3 months)
- No change
- Moderate delays (1-3 months)
- Major delays (>3 months)

  [Free text for additional context]

(2) If delayed, by approximately how many months?
- X months delay on overall access timelines

(3) If reimbursement is likely to be delayed, do you see opportunities to shape the system to minimize delay?
- Yes
- No

- Maybe
  [Free text for additional context]

JCA process /national process dependencies:

(4) Do you have the option to seek advice from your HTA body to inform Phase 3 (pivotal) study design (and if yes, at what timepoint relative to which EMA submission?)
- Yes
- No
  [free text for additional context]

(5) Do you have the option to seek advice from your HTA body about the PICOs of relevance for the national HTA process? (and if yes, at what timepoint relative to which EMA milestone?)
- Yes
- No
  [free text for additional context]

(6) How do you anticipate your national HTA body will deal with new data cuts which become available after publication of the JCA report
- Request updated analysis during national submission
- Trigger a JCA reassessment but proceed with national submission
- Trigger a JCA reassessment and pause national process while awaiting an updated JCA report
- Currently uncertain
- Not relevant

Survey 2: Issued via MS Forms in second half of 2025

Survey issued to local affiliates representing the following markets (n=25)	Responses received from local affiliates representing the following markets (n=19)	Participation rate
Austria<br>Belgium<br>Bulgaria<br>Croatia<br>Czechia<br>Denmark<br>Estonia<br>Finland<br>France<br>Germany<br>Greece/Cyprus/Malta<br>Hungary<br>Ireland<br>Italy<br>Latvia<br>Lithuania<br>Netherlands<br>Norway<br>Poland	Austria<br>Belgium<br>Bulgaria<br>Croatia<br>Cyprus<br>Czechia<br>Estonia<br>France<br>Germany<br>Ireland<br>Italy<br>Netherlands<br>Norway<br>Poland<br>Romania<br>Slovakia<br>Slovenia<br>Spain<br>Sweden	19/25 (76%)

Portugal<br>Romania<br>Slovakia<br>Slovenia<br>Spain<br>Sweden		

<u>Context and questions:</u>

MSD has agreed to review our position paper to incorporate today's collective intelligence and prioritize key challenges accordingly for productive discussions at EU level moving forward.

Please find below a short survey with the following content:

- *Pre-populated positions/requests will be included for you to rank appropriately based on relevance to your local situation*

Responses to the survey are requested by 1st December 2025

1. Country: [Enter response]

2. Please rank the following topics in order of importance/relevance from your national perspective for the updated MSD position paper.

   - Submission of additional data packages to the EMA and associated notification obligations
   - Transparency criteria for PICO consolidation
   - Consideration of the JCA report in national appraisals and the impact of delays in the JCA process
   - European Commission support for patient organisations
   - Systematic stakeholder feedback, including industry input, on the JCA process to inform its evaluation by 2028
   - Development of a dedicated JCA process for vaccines
